# Supplementary material for: Physical properties of the HIV-1 capsid from all-atom molecular dynamics simulations
Source: Nat Commun. 2017 Jul 19;8:15959. doi: 10.1038/ncomms15959 (PMC5524983; doi:10.1038/ncomms15959)
Supplement: Supplementary Information [file ncomms15959-s1.pdf]

Type of file: PDF
Title of file for HTML: Supplementary Information
Description: Supplementary Figures, Supplementary Notes and Supplementary References.

Type of file: MP4
Title of file for HTML: Supplementary Movie 1
Description: All-atom simulation in a coarse-grained representation. Each bead represents a hexamer o pentamer. Hexamers are shown in tan and pentamers in green.

Type of file: MP4
Title of file for HTML: Supplementary Movie 2
Description: Translocation of chloride ions through hexamers. Chloride ions are shown in Cyan and Arginine 18 is shown in licorice representation.

Type of file: MOV
Title of file for HTML: Supplementary Movie 3
Description: Translocation of ions through the HIV-1 capsid. Left: Chloride ions (cyan) translocate through the center of hexamers. Right: Sodium ions (yellow) translocate through the seams between hexamers (right).

Type of file: MP4
Title of file for HTML: Supplementary Movie 4
Description: HIV-1 capsid fluctuations. Fluctuations on the surface ranging from -3 to +3 Å (red to blue).

Type of file: ZIP
Title of file for HTML: Supplementary Data 1
Description: The first 100 PCs included , can be visualized using the NormalModeWizard available in VMD.

Type of file: PDF
Title of file for HTML: Peer Review File
Description:

Supplementary note 1

Locations of the pentamers, given in terms of IUPAC's canonical ring spiral pentagon indices (RSPI), were previously determined using the cross-correlation between a segmented HIV-1 core acquired by cryo electron-tomography and candidate fullerene models.[1] The RSPI for the structure in the present study were: 1, 8, 21, 62, 64, 90, 136, 165, 199, 204, 215, and 227. The selected geometry-optimized polyhedral-cage served as a template for building an atomistic model. The CA hexamer derived from molecular dynamics flexible fitting (MDFF) and equilibrated CA pentamer replaced hexagons and pentagons in the cage structure, respectively. [1] For mapping into the cage, the centers of mass at the threefold symmetry axes were computed from the hexamer of hexamers and pentamer of hexamers simulations,[1] resulting in six and five possible centers of mass for each oligomer, respectively; the resulting center of mass rings were aligned to the hexagons and pentagons in the polyhedral cage. Given the inherent curvature of the MDFF-derived hexamer, all six possible orientations of the all-atom model were fitted to each hexagon of the polygonal cage and the best fit, based on RMSD, was selected. The first solvation shell from simulation, including water and ions were kept during the modeling of hexamers and pentamers;[1] conflicting water and ions between neighboring hexamers and pentamers were removed. The resulting structure, comprising the all-atom HIV-1 virus like particle, together with the first hydration shell, included a total of 11,640,222 atoms. Additionally, bulk water and ions were added to the structure, setting the ion concentration to 150 mM NaCl. The fully solvated and ionized structure contained a total of 64,423,983 atoms.

Supplementary note 2

The simulation and analysis of multi-million atom systems requires specialized software and hardware.[2] Indeed, NAMD, through the use of the so-called memory-optimized builds,[3] GPU computing,[4] and topology-aware load-balancing,[5] efficiently harnesses the power of Petascale supercomputers, thus providing the computing power necessary to simulate the HIV-1 VLP in all-atom detail. Notably, NAMD performance on 3880 Titan XK7 nodes sustains an outstanding 8 $\pm$ 2 ns/day for the 64 million atom simulation. In addition, for all simulations above the 10 million atom threshold, the recently released JS (John Stone) file-format[6] supersedes the old protein structure file (PSF), as it has virtually no limitations with respect to both atom count and

number of coordinates. The JS file-format is available in psfgen, VMD and NAMD.

Analysis of multi-million molecular dynamics trajectories also posed a serious challenge for the present study. Notably, the size of a trajectory file is 315 Gbytes for every 8 ns of simulation. Taking advantage of the capabilities of the Lustre file-system, the electrostatic potential of the capsid was calculated in parallel for each trajectory frame. Calculation of the all-atom electrostatics is also memory-intensive as it uses over 100 Gbytes of the 128 Gbytes of physical memory available per node in OLCF's Rhea commodity cluster.

Due to the large number of degrees of freedom, analyses like PCA that depend on linear-algebra operations constitute another computational challenge. Our analysis took advantage of highly optimized linear algebra libraries (Intel Math Kernel Library), in particular the multi-core subroutines. All linear-algebra calculations were performed on an Intel XEON E5-2643 workstation equipped with 512 GBytes of physical memory.

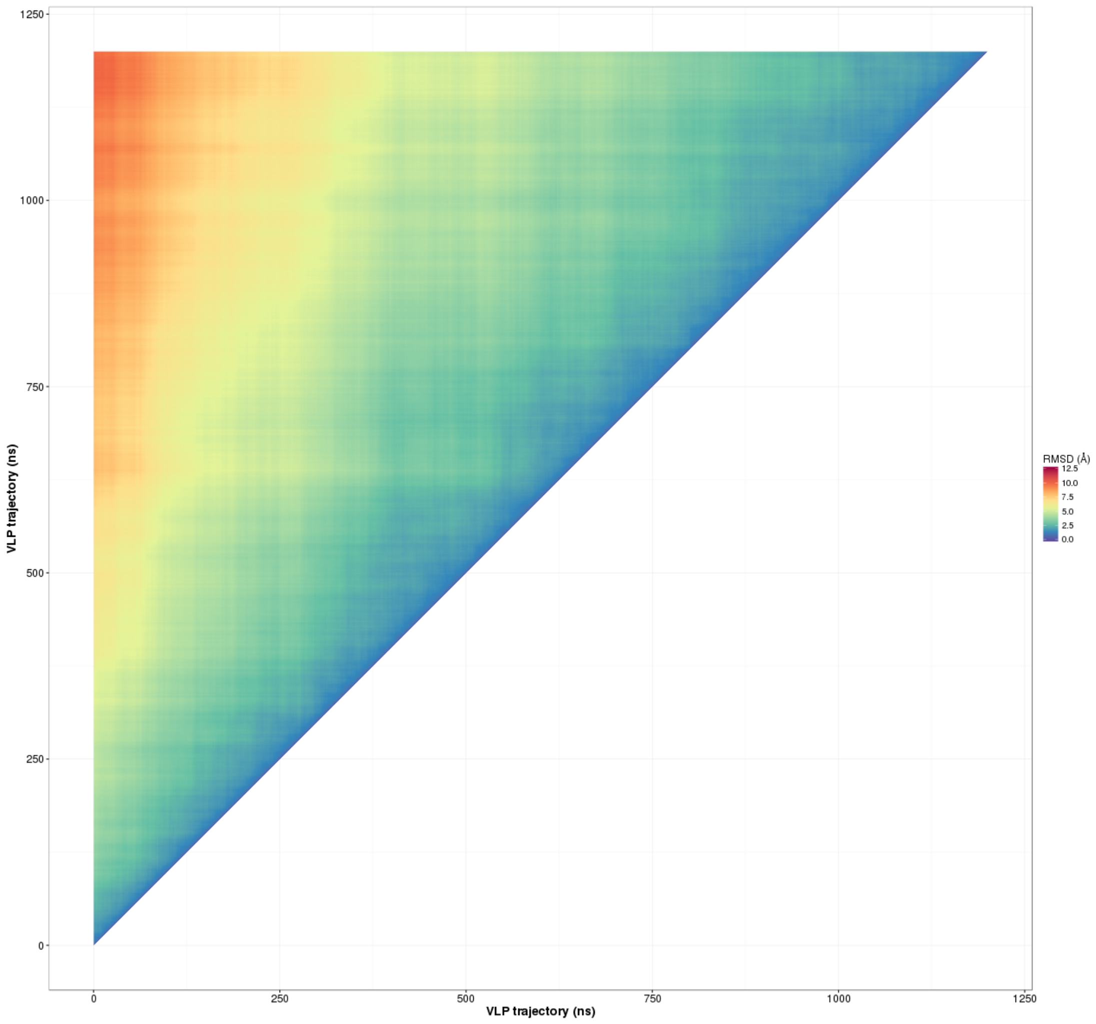

Supplementary Figure 1: Conformational sampling characterized by the RMSD-based distance-matrix. Each element of the matrix represents the RMSD between entire capsid strucures at given simulation time-points. RMSD was calculated using a coarse-grained time series, employing a bead for the center of mass of each pentamer/hexamer (Movie M1). The figure demonstrates that the simulation reaches a stable configuration after 400 ns.

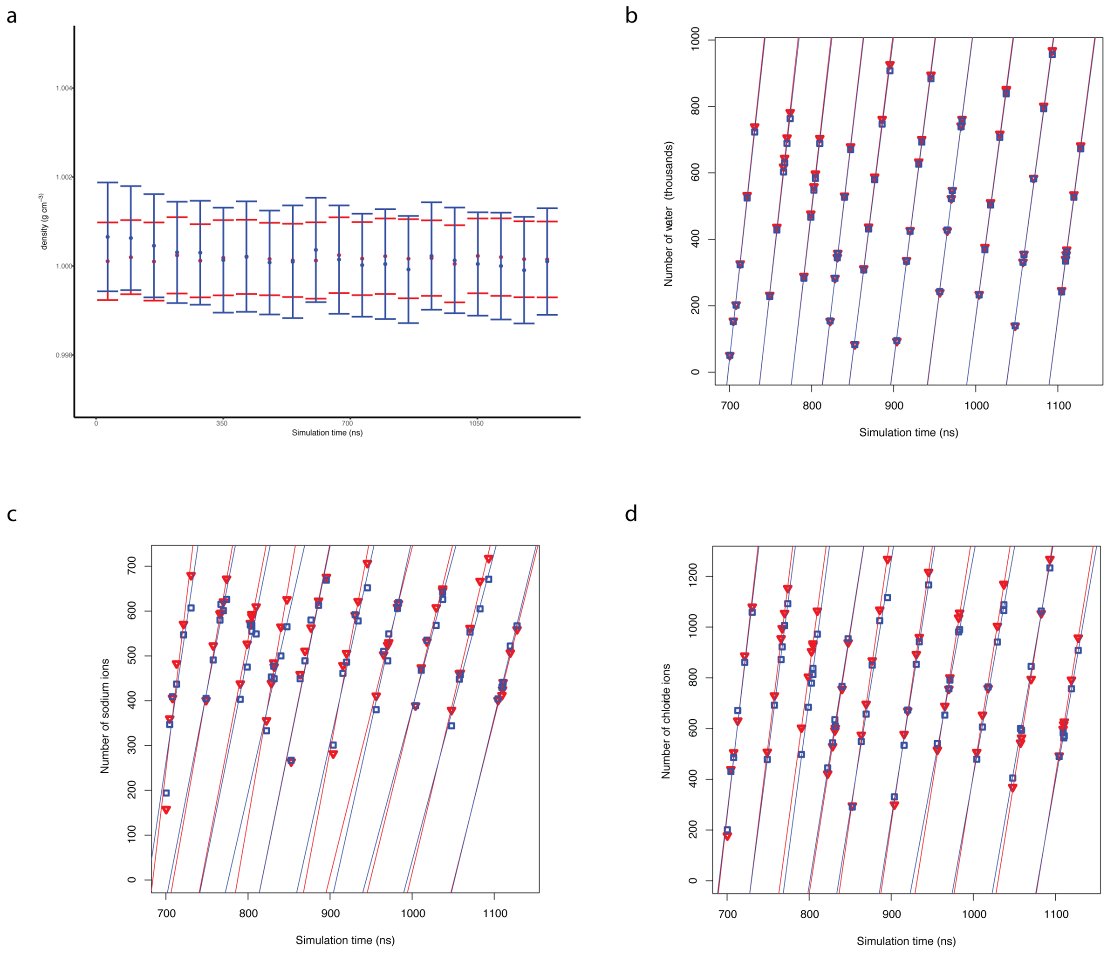

Supplementary Figure 2: Density and exchange rates of water and ions. a) Block-averaged water density inside (blue) and outside (red) of the capsid in $\mathrm{gm} \cdot \mathrm{cm}^3$ over the course of the simulation. The initial difference between the inside and outside of the capsid subsides during the first 200 ns of the simulation. b) Cumulative number of waters crossing the surface of the capsid going outwards (red) and inwards (blue). The linear fit highlights the convergence between the two rates over segments of the trajectory, and the slope determines the exchange rate going out and in to be $20.1 \times 10^3 \pm 1.6 \times 10^3$ and $19.7 \times 10^3 \pm 1.5 \times 10^3$ water molecules per ns, respectively. c) Cumulative number of sodium ions crossing the surface of the capsid going outwards (red) and inwards (blue). Overall, the same number of sodium ions are going out and in with rates 9.4 ± 2.4 and 8.5 ± 1.1 sodium molecules per ns, respectively. d) Cumulative number of chloride ions crossing the surface of the capsid going outwards (red) and inwards (blue). Chloride translocates out and in at a rate of 22.2 ± 2.9 and 20.8 ± 3.3 chloride molecules per ns, respectively.

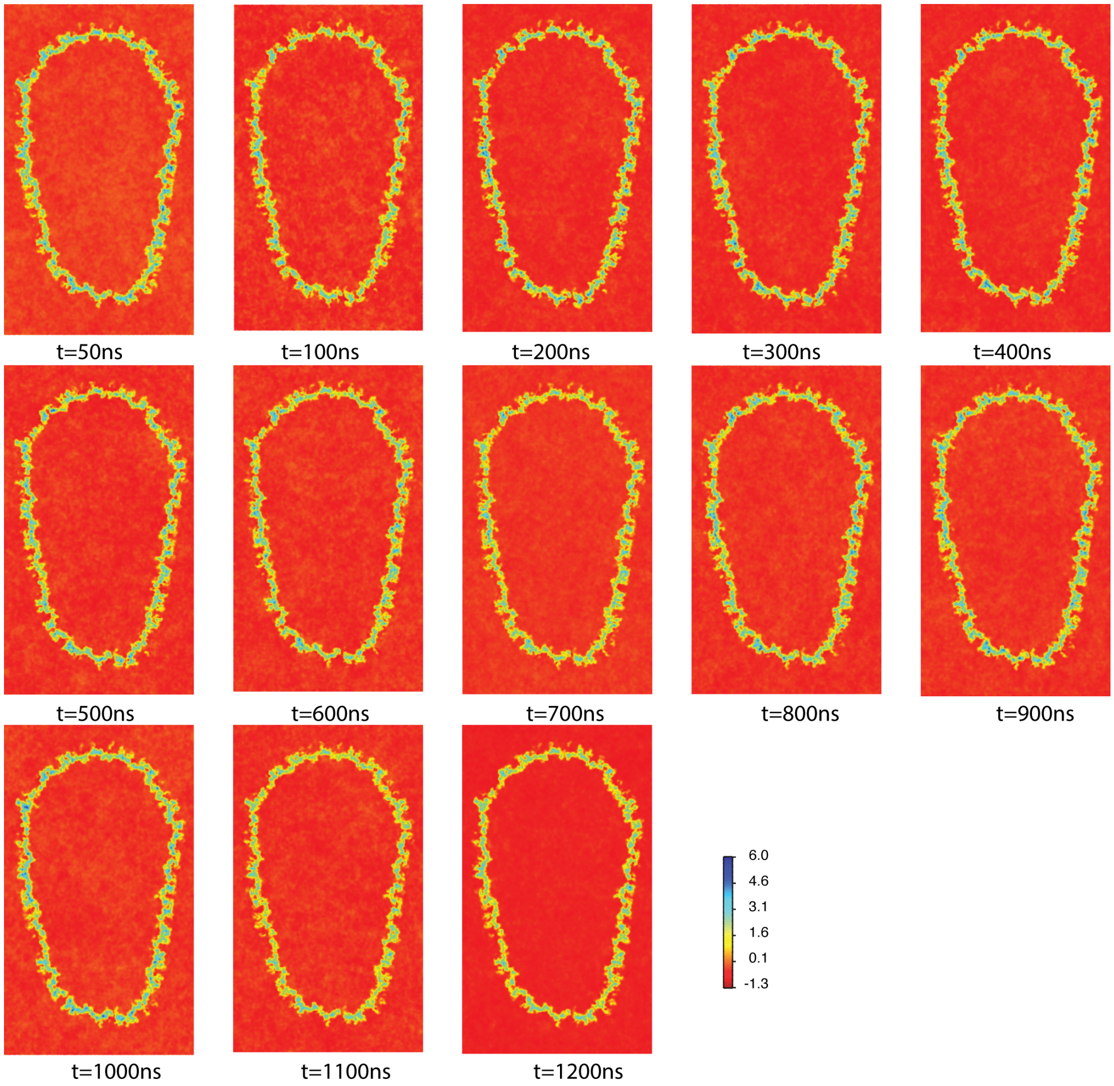

Supplementary Figure 3: Electrostatics time-lapse over the course of the simulation. Snapshots captured at different time-points during the simulation showing the average electrostatic potential over short fragments of the simulation; the average length of each fragment is 10 ± 4 ns. Similar characteristics can be observed for the different snapshots, in particular, the interior and exterior of the capsid remain at the same electrostatic potential throughout the simulation.

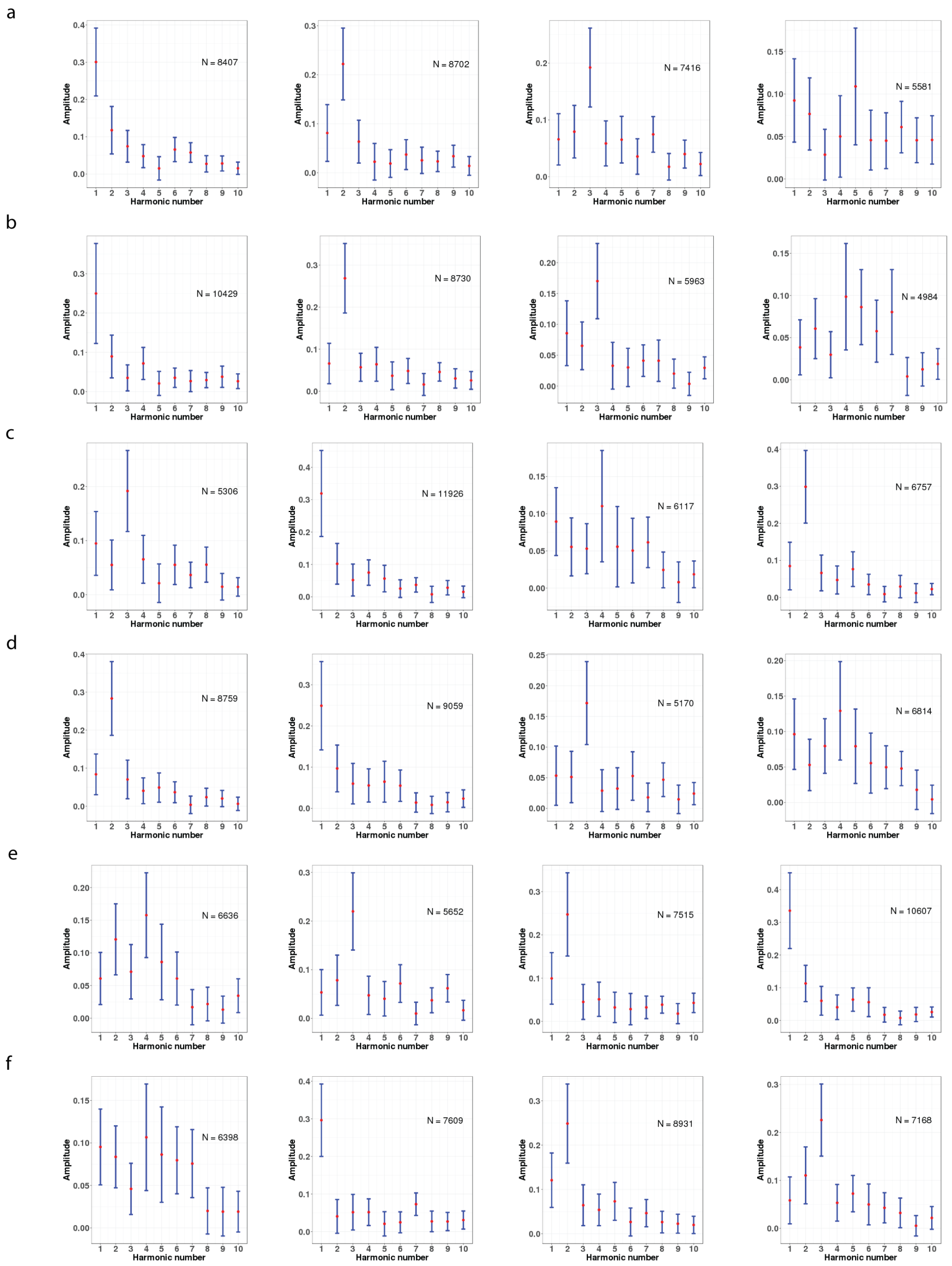

Supplementary Figure 4: Periodograms of the time series of the capsid motions. The red dots denote the medoid of the cluster and the error bars represent the variability within each cluster. a-b) Four classes of periodograms were found for a 400 ns trajectory split into two 200 ns trajectories. Similar dominant clusters are found between the two halves of the trajectory. c-f) Four classes of periodograms were found for the 400 ns trajectory split into four 100 ns trajectories. Similar dominant harmonics where found for the four segments of the trajectory.

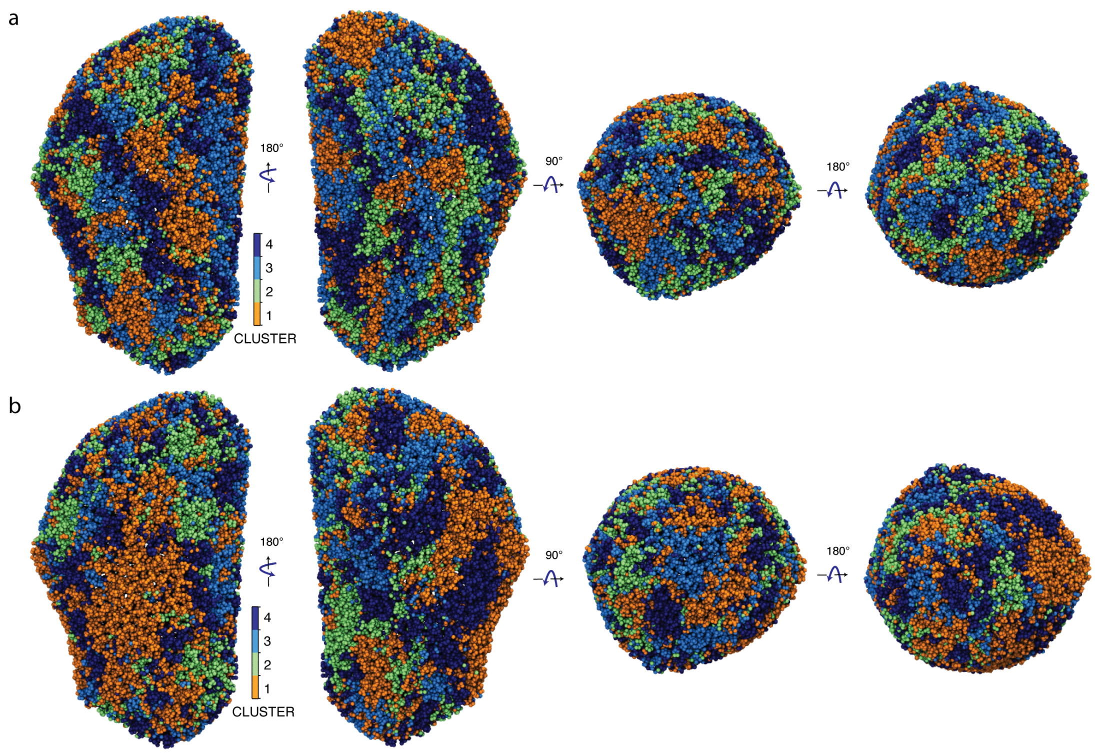

Supplementary Figure 5: Projection of the acoustic clusters onto the structure of the capsid. A single 400 ns trajectory was split into two 200 ns trajectories. Since the fundamental frequencies found in Figure S4 match between the two halves of the trajectory, direct comparison of the projections is possible. a) Projection of the first half of the trajectory onto the surface of the capsid, indicating the location of each cluster. b) Projection of the second half of the trajectory onto the surface of the capsid, indicating the location of each cluster.

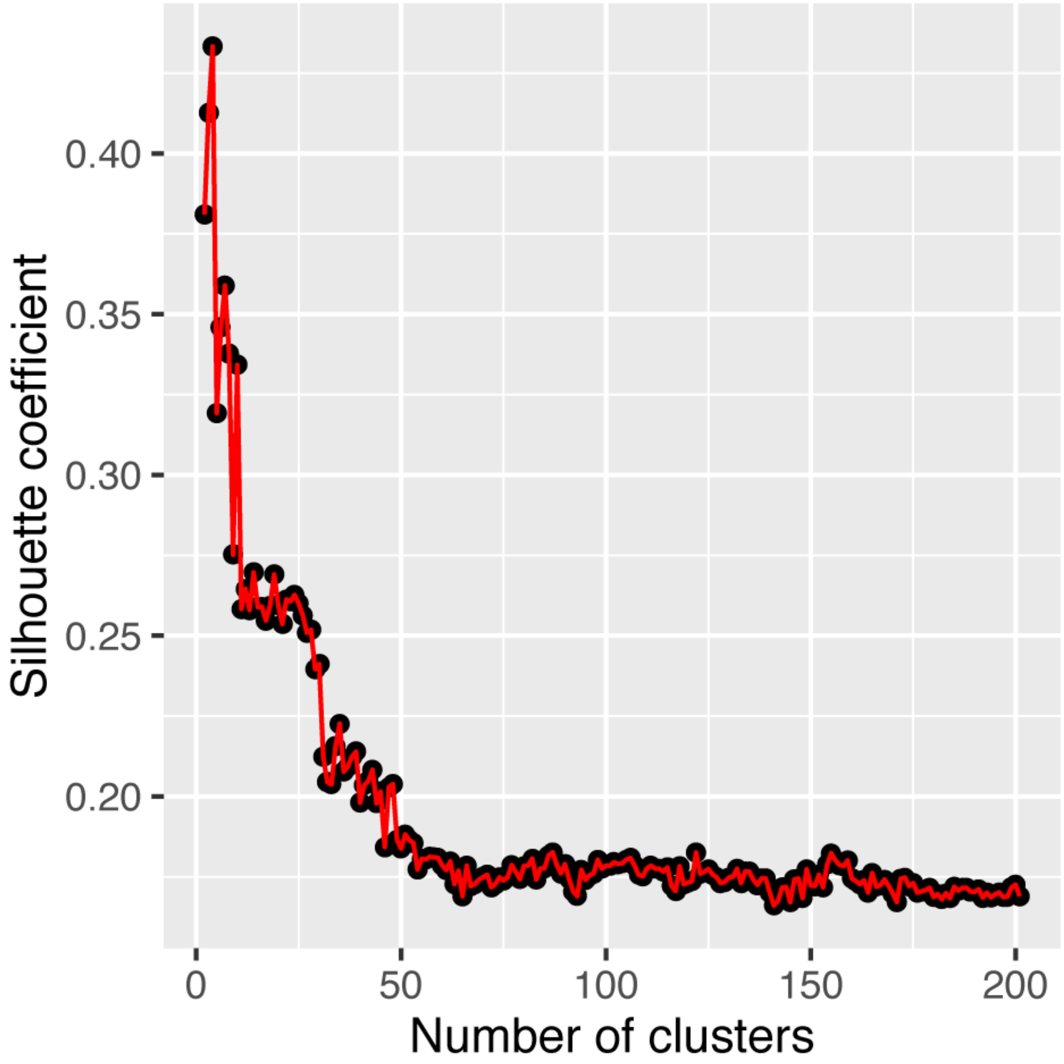

Supplementary Figure 6: Silhouette coefficient for acoustic cluster numbers from $k = 2 \ldots 200$. A peak can be observed for $k = 4$, indicating the optimal number of clusters.

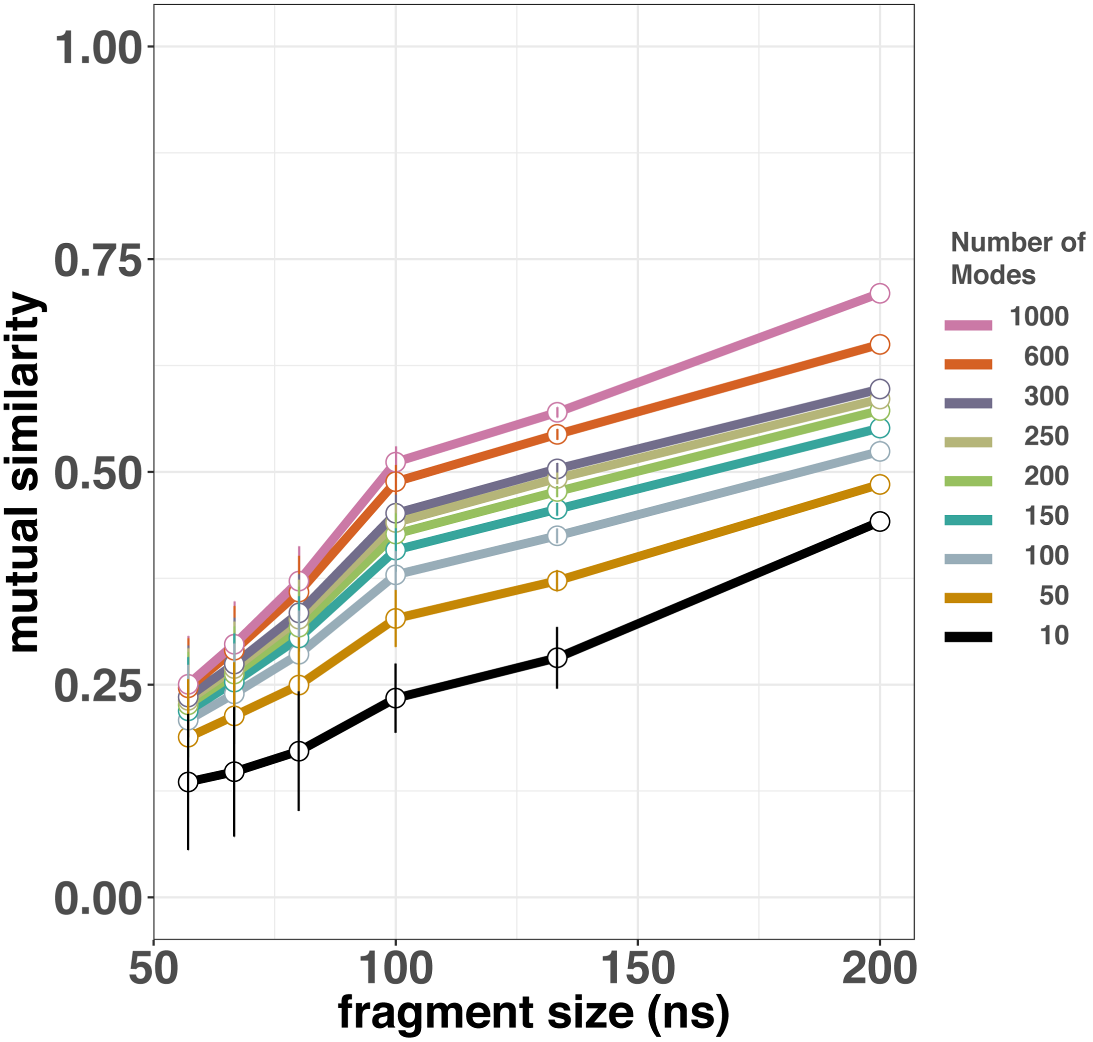

Supplementary Figure 7: Convergence of conformational subspaces spanned by principal components (PCs) for the HIV-1 capsid. The 400 ns trajectory was divided into multiple fragments of same length varying from 50 ns to 200 ns; subsequently principal component analysis was performed on each fragment. The lines show the mutual similarity between PCs corresponding to different fragments of the trajectory as a function of fragment size. Different dimensionalities of the PC-subspaces from 10 to 1000 modes are shown in different colors.

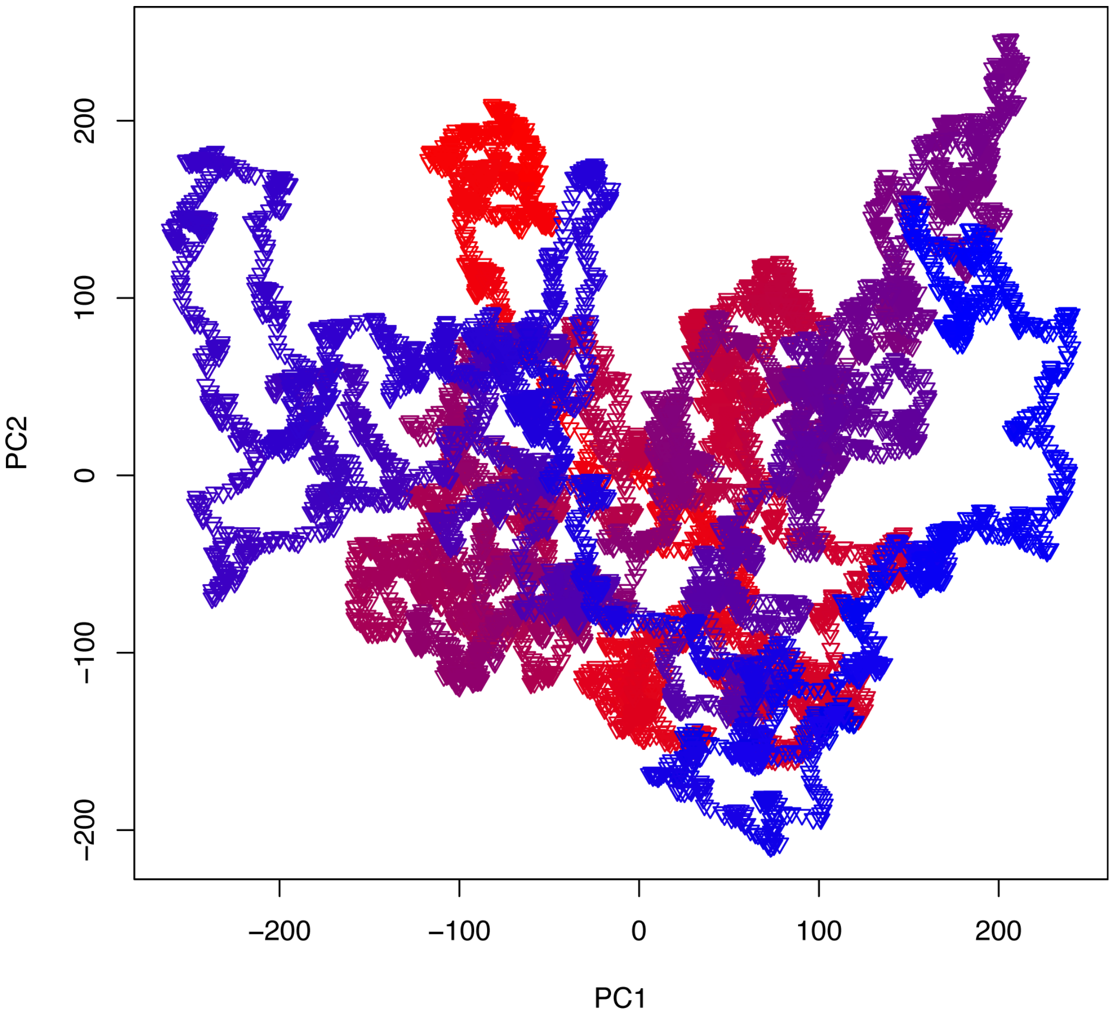

Supplementary Figure 8: Projection of the last 400 ns of the trajectory onto the first two principal components. Time evolution is presented as a color gradient of the trajectory going from red (0 ns) to blue (400 ns).

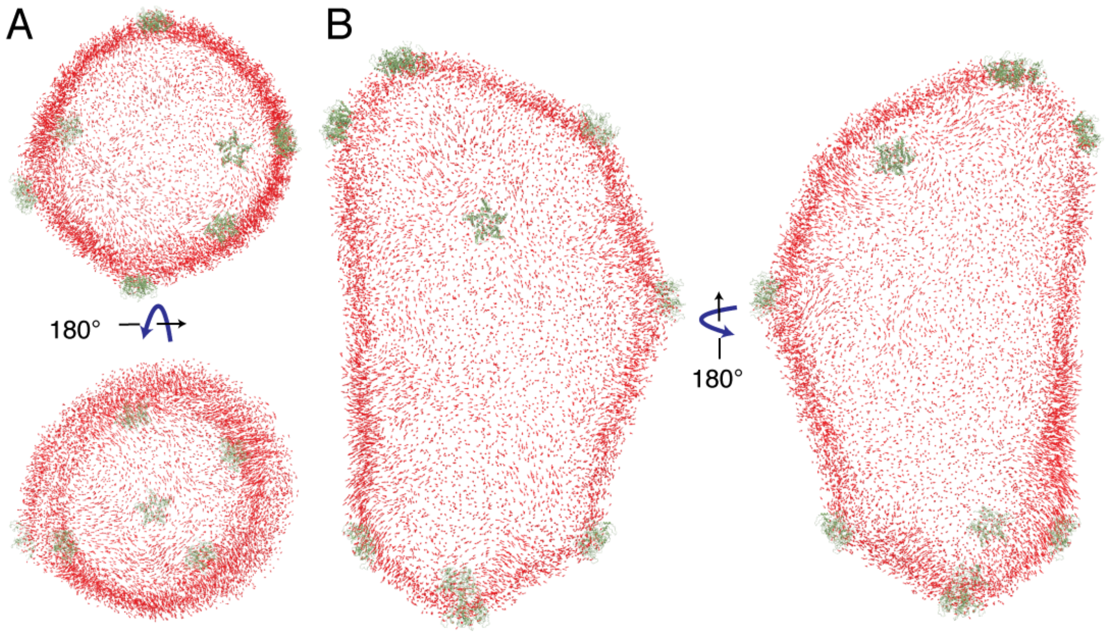

Supplementary Figure 9: Mechanical motions of the HIV-1 capsid. Projection of the displacement patterns for the second PC. The direction of the displacement is represented by the direction of the arrow; similarly, the magnitude of the displacement is represented by the length of the arrow. Locations of pentamers are highlighted in green.

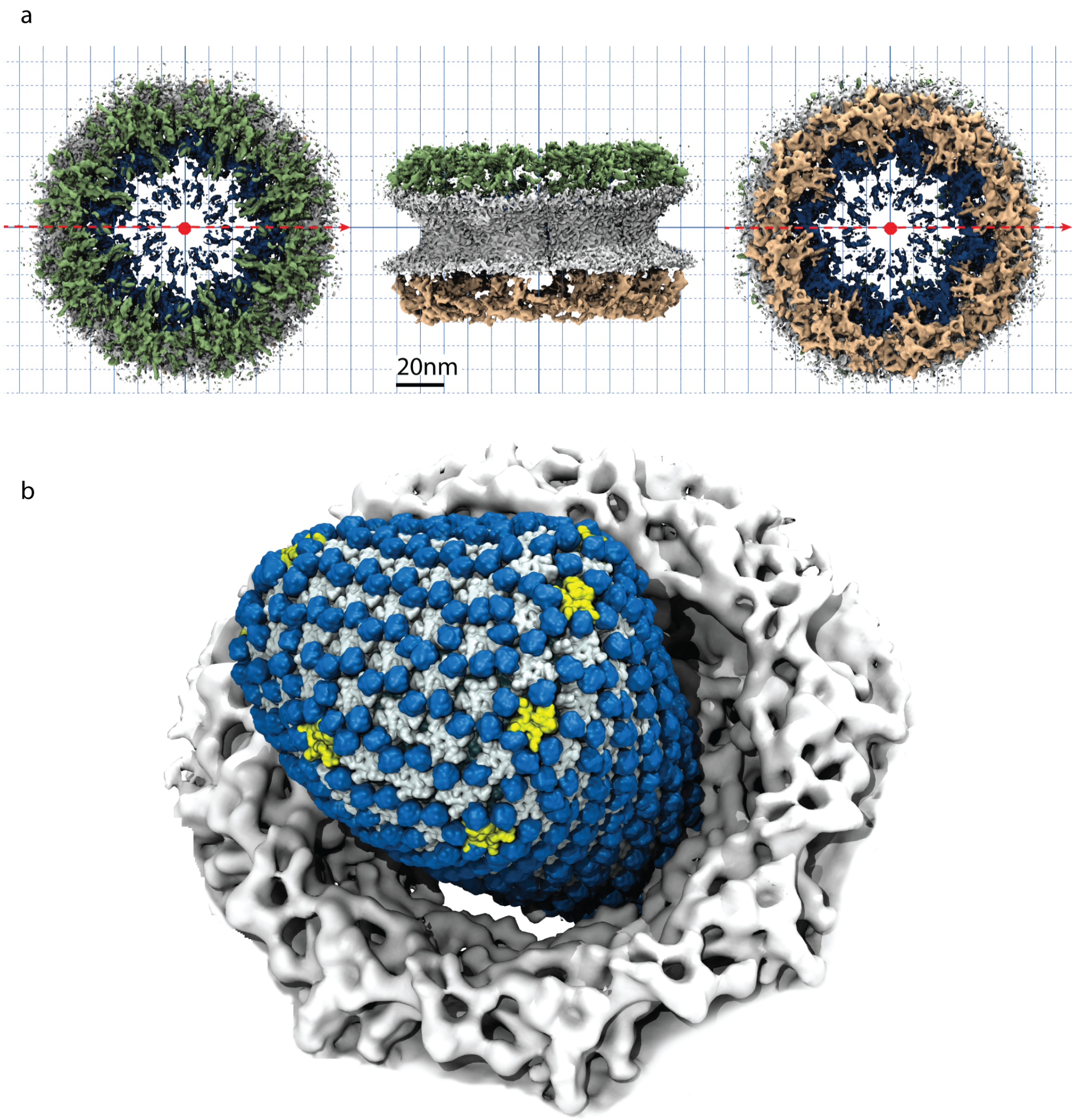

Supplementary Figure 10: Capsid of the human immunodeficiency virus type-1 (HIV-1) docked to the nuclear pore complex (NPC) during HIV infection; docking performed to scale. a) Experimental cryo electron-tomography density of the Xenopus Laevis NPC .[7] b) The HIV-1 VLP computer model generated in the present study docked to the density of the NPC. The capsid is decorated with Cyclophilin-A[8] (shown in blue).

Supplementary References

(1) Zhao, G.; Perilla, J. R.; Yufenyuy, E. L.; Meng, X.; Chen, B.; Ning, J.; Ahn, J.; Gronenborn, A. M.; Schulten, K.; Aiken, C.; Zhang, P. *Nature* **2013**, *497*, 643–646.

(2) Perilla, J. R.; Goh, B. C.; Cassidy, C. K.; Liu, B.; Bernardi, R. C.; Rudack, T.; Yu, H.; Wu, Z.; Schulten, K. *Current Opinion in Structural Biology* **2015**, *31*, 64–74.

(3) Mei, C.; Sun, Y.; Zheng, G.; Bohm, E. J.; Kalé, L. V.; Phillips, J. C.; Harrison, C. Enabling and Scaling Biomolecular Simulations of 100 Million Atoms on Petascale Machines with a Multicore-optimized Message-driven Runtime. Proceedings of the 2011 ACM/IEEE conference on Supercomputing. Seattle, WA, 2011; pp 61:1–61:11.

(4) Stone, J. E.; Phillips, J. C.; Freddolino, P. L.; Hardy, D. J.; Trabuco, L. G.; Schulten, K. *Journal of Computational Chemistry* **2007**, *28*, 2618–2640.

(5) Phillips, J. C.; Sun, Y.; Jain, N.; Bohm, E. J.; Kalé, L. V. Mapping to Irregular Torus Topologies and Other Techniques for Petascale Biomolecular Simulation. Proceedings of the International Conference on High Performance Computing, Networking, Storage and Analysis. 2014; pp 81–91.

(6) Stone, J. E.; Vandivort, K. L.; Schulten, K. *Lecture Notes in Computer Science* **2011**, *6939*, 1–12.

(7) Eibauer, M.; Pellanda, M.; Turgay, Y.; Dubrovsky, A.; Wild, A.; Medalia, O. *Nature communications* **2015**, *6*, 7532.

(8) Liu, C. et al. *Nature Communications* **2016**, *7:10714*, (10 pages).
